# Supplementary material for: An encrusting kleptoparasite-host interaction from the early Cambrian
Source: Nat Commun. 2020 Jun 2;11:2625. doi: 10.1038/s41467-020-16332-3 (PMC7266813; doi:10.1038/s41467-020-16332-3)
Supplement: Supplementary file 1 — Supplementary Information [file 41467_2020_16332_MOESM1_ESM.pdf]

Supplementary Information
An encrusting kleptoparasite-host interaction from the early Cambrian

Zhang et al.

This file comprises:

Supplementary Fig. 1: Locality map and stratigraphic position of the Guanshan Konservat-Lagerstätte in Yunnan Province.
Supplementary Fig. 2: Additional examples of *N. wulongqingensis* from the Guanshan biota encrusted with varying numbers of obligate, kleptoparasitic tube-dwelling organisms
Supplementary Fig. 3: Exceptionally preserved examples of *N. wulongqingensis* from the Guanshan biota with chaetae preserved together with morphological details of encrusting biomineralised tubes.
Supplementary Fig. 4: The basibiont neobolid (Lingulata) brachiopod *Neobolus wulongqingensis* from the Guanshan biota (Cambrian Stage 4) of eastern Yunnan.
Supplementary Fig. 5: Aggregations of *Neobolus wulongqingensis* with associated attached obligate kletoparasitic tube-dwelling organisms, showing the density of individuals per unit area.
Supplementary Fig. 6: Supplementary plots exploring relationship between brachiopod biomass and characteristics of attached tubes.
Supplementary Fig. 7: Additional rose diagrams of attached tube orientation for all values of attached tubes.
Supplementary Fig. 8: Diagram showing methods used to take measurements of encrusted tubes.

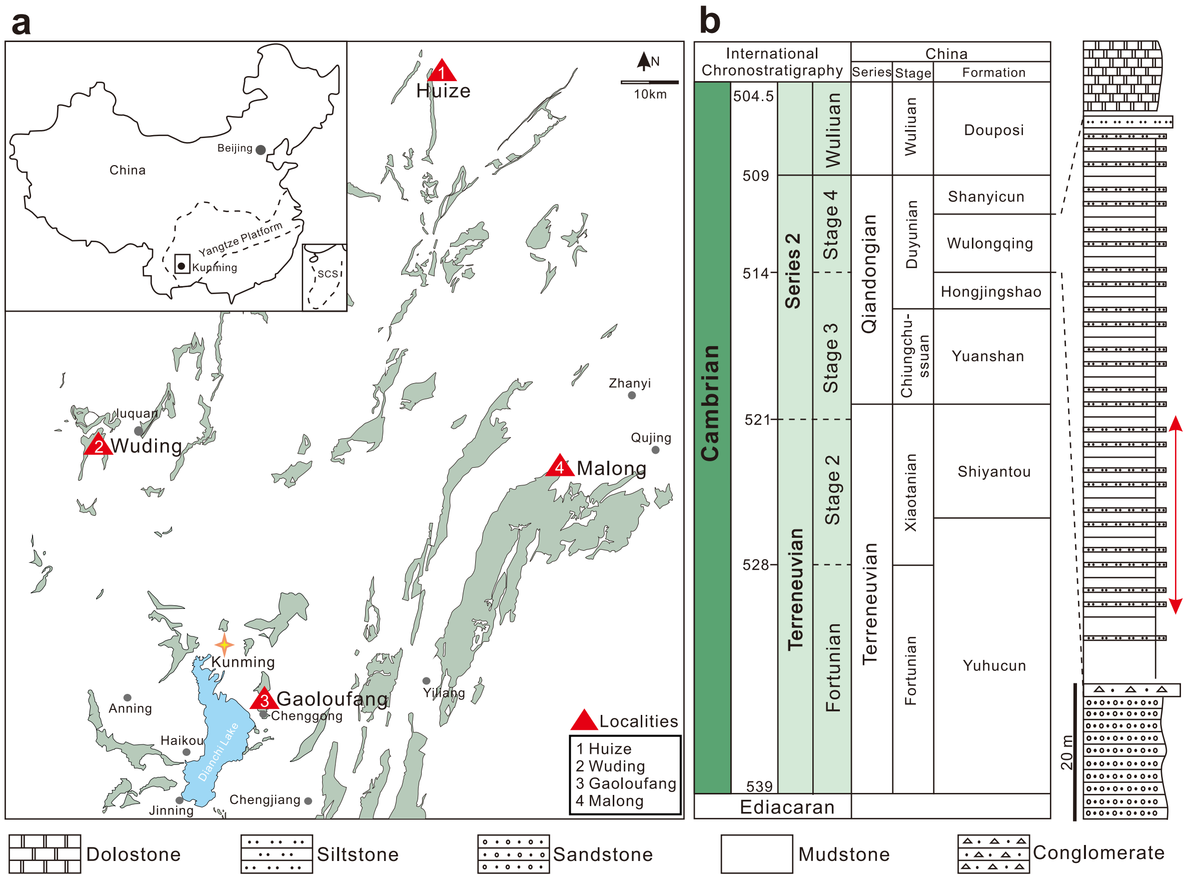

Supplementary Fig. 1. Locality map and stratigraphic position of the Guanshan Konservat-Lagerstätte in Yunnan Province. **a,** Locality map for the Guanshan Konservat-Lagerstätte in Yunnan Province (modified from[1]). All specimens of *Neobolus wulongqingensis* included in this study were sampled from the Gaoloufang section (Locality 3). **b**, Cambrian stratigraphic scheme, both international and for China, showing the relative position of the Wulongqing Formation.

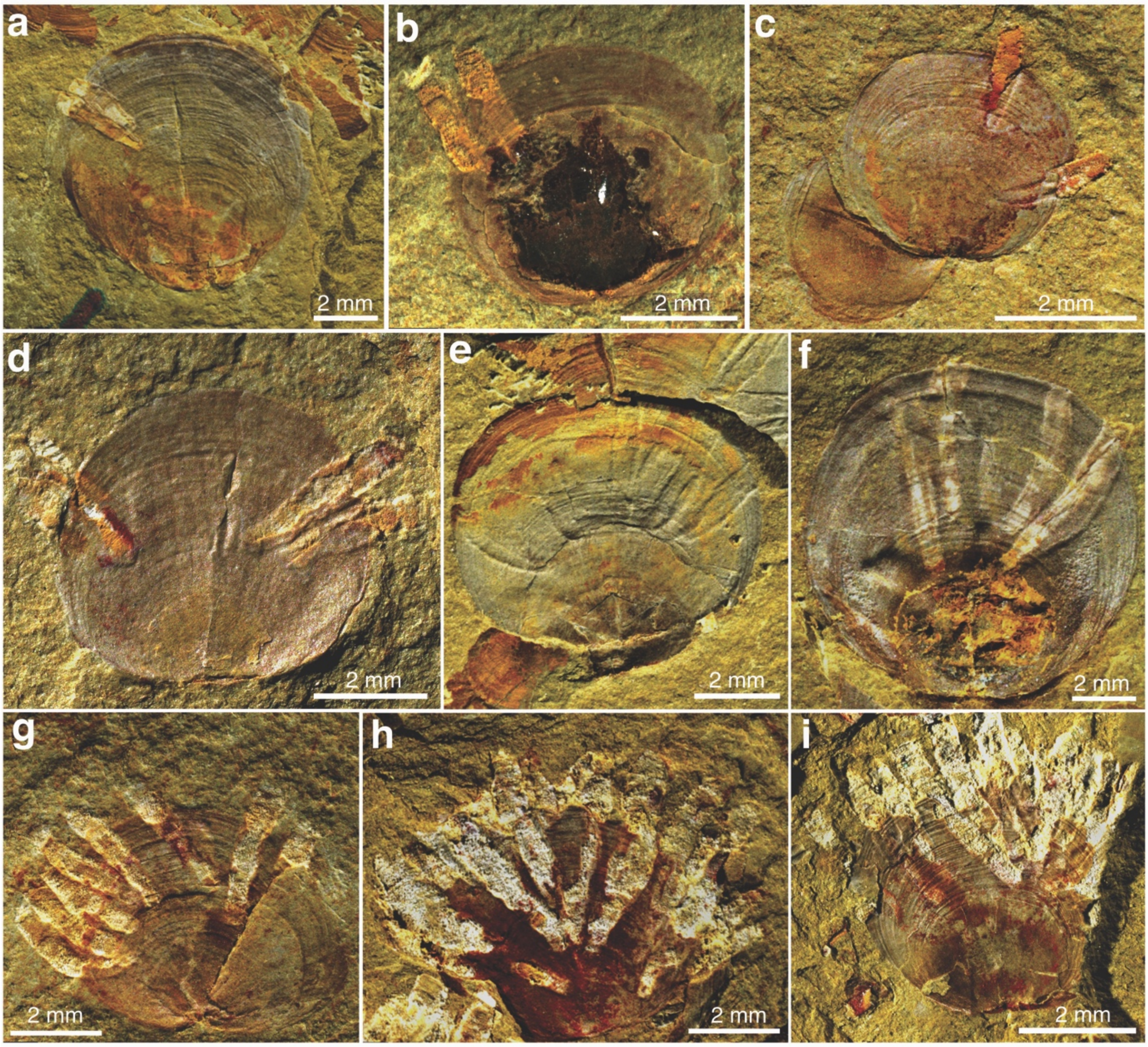

Supplementary Fig. 2. Additional examples of *N. wulongqingensis* from the Guanshan biota encrusted with varying numbers of obligate, kleptoparasitic tube-dwelling organisms (see Supplementary Dataset 1 for further details). a, ELI GB-N- 263A5-1. **b,** ELI GB-N-254-1-1, *N. wulongqingensis* with visceral region preserved **c,** ELI GB-N-253-19-1-D2, *N. wulongqingensis* with tube outlines visible from the interior of the brachiopod shell **d,** ELI GB-N-290-8-1. **e,** ELI GB-N-263A-2-3, *N. wulongqingensis* with the outline of the kleptoparasitic tubes visible from the interior of the brachiopod shell **f,** ELI GB-N-0263B-3. **g,** ELI GB-N-0253. **h,** 284-3-1. **i,** ELI GB-N-0255-8.

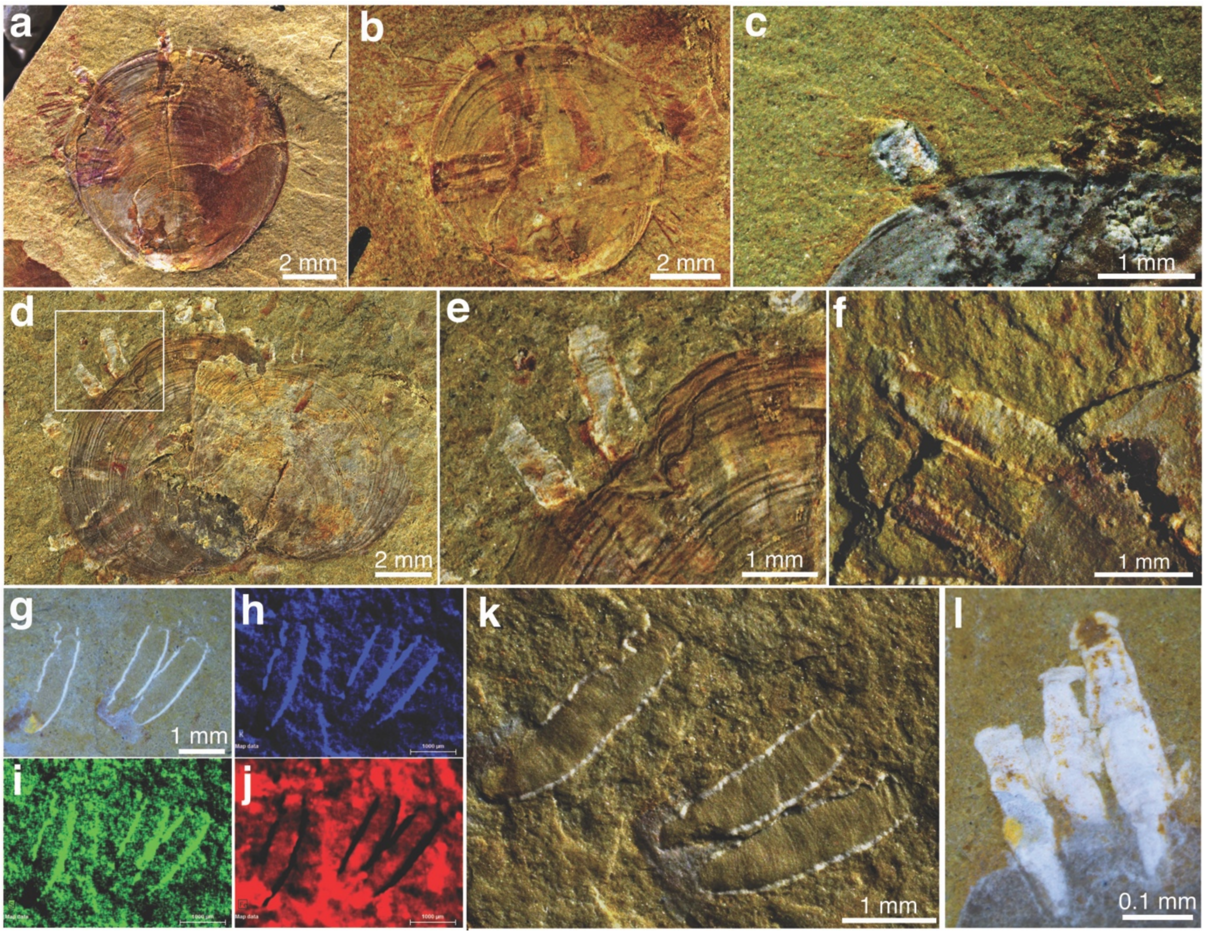

Supplementary Fig. 3. Exceptionally preserved examples of *N. wulongqingensis* from the Guanshan biota with chaetae preserved together with morphological details of encrusting biomineralised tubes. a, b, exceptionally preserved specimens of *N. wulongqingensis* with chaetae preserved: **a**, ELI GB-N 632-1. **b**, 648-6-1-GLF. **c,** ELI GB-N 561-2-1, close up of kleptoparsitic tube extending into the chaetal fringe of a *N. wulongqingensis* individual. **d, e,** ELI GB-N 258-1-1. **d,** twisted dorsal and ventral valves with encrusting kleptoparasitic tubes. **e,** enlarged view of boxed area in Supplementary Fig. 3d. **f,** 258 -1-2 –GLF, Close up of biomineralised tubes showing clear surface annulations**. g, h,** 250 -1 -1-2- GLF. Detail and micro-XRF elemental mapping of biomineralised tubes detached from brachiopod valve. **g,** light photo. **h,** Micro-XRF elemental mapping of K. **i,** Micro-XRF elemental mapping of Si. **j,** Micro-XRF elemental mapping of Fe. **k,** Close up of the three biomineralised tubes. **l,** close up of kleptoparasitic tubes still attached to *N. wulongqingensis*.

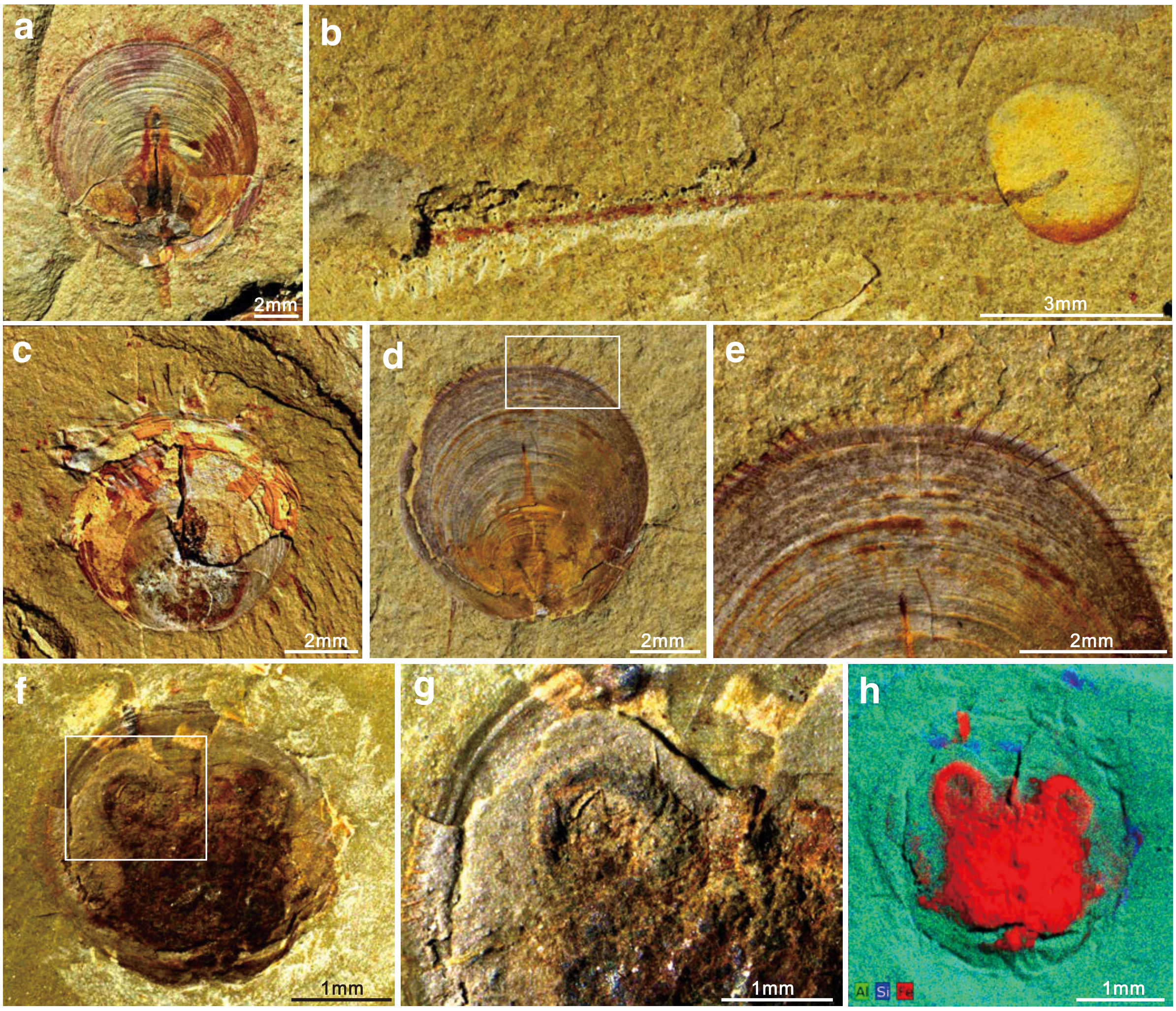

Supplementary Fig. 4. The basibiont neobolid (Lingulata) brachiopod *Neobolus wulongqingensis* from the Guanshan biota (Cambrian Stage 4) of eastern Yunnan. a, Holotype, ELI GB-N-0377-1, a composite mould with dorsal and ventral valves strongly compressed. Note the fringe of chaetae and proximal pedicle. **b,** ELI GB-N-0297-4, a 3-dimensional ventral valve with a preserved elongated pedicle attached to an exoskeleton of a trilobite. **c,** ELI GB-N-0625, compressed dorsal and ventral valves, showing the dorsal and ventral chaetae cross to form a fine sieve or mesh. **d, e,** ELI GB-N-0385: **d,** View of dense anterior marginal chaetae; **e,** Magnified view of boxed area in Fig. S4D. **f, h,** ELI GB-N-SJJ-1308, shell interior, showing paired spiral lophophore: **f,** light photograph; **g,** enlarged view of boxed area in Supplementary Fig. 4f; **h,** Micro-XRF elemental mapping of Al, Si and Fe shows paired spiral lophophore in high contrast.

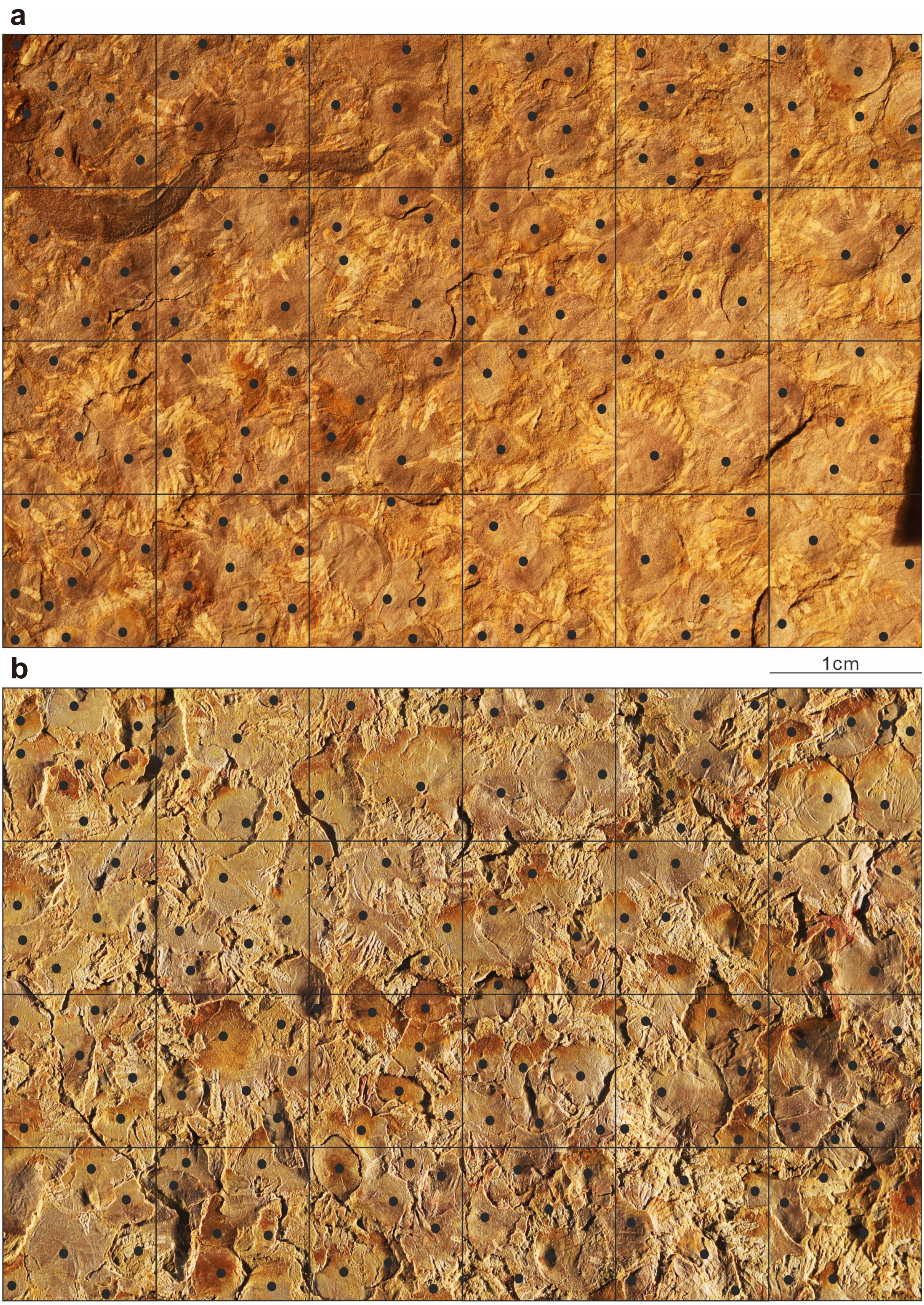

Supplementary Fig. 5. Aggregations of *Neobolus wulongqingensis* with associated attached obligate kletoparasitic tube-dwelling organisms, showing the density of individuals per unit area. Each square equals 1 cm^2 and each black dot equals one individual brachiopod. **a,** ELI GB-N-N-0300. **b,** ELI GB-N-N-0301.

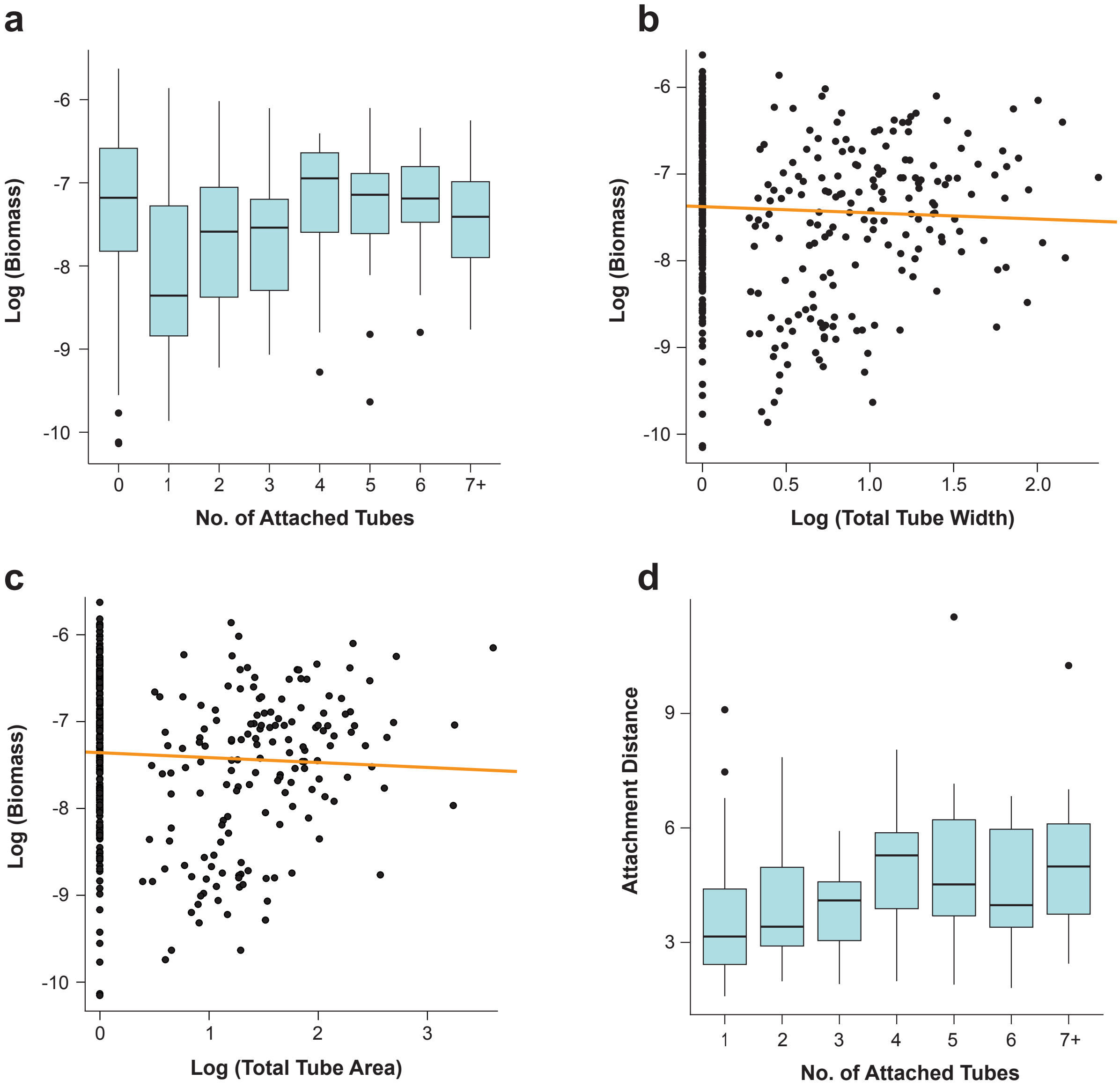

Supplementary Fig. 6. Supplementary plots exploring relationship between brachiopod biomass and characteristics of attached tubes. a, Box and whisker plot of number of attached tubes versus Brachiopod biomass. Whilst 1-3 attached tubes results in lower median biomass compared to individuals without tubes, individuals with 4+ tubes are indistinguishable from those with no attached tubes. **b,** Plot of total tube width versus biomass. Total tube width for each individual is calculated as the sum total width of all tubes present on the relevant individual. **c,** Plot of total tube area versus biomass. Total tube area for each individual is calculated as the sum total area of the shell surface covered by the attached tubes for the relevant individual. **d,** Box and whisker plot of number of attached tubes versus attachment distance. Increasing the number of tubes per individual results in an increase in attachment distance. With attachment distance representing a proxy for time of attachment, this result suggests that large numbers of parasites are present for shorter durations and are only possible on larger, older shells. Number of biologically independent specimens used for each plot: 6a = 429; 6b =408; 6c = 383; 6d = 167. Data used to construct Supplementary Figures 6a-d provided in Supplementary Data 1 and 2. Box and whisker plot results provided in Supplementary Data 3.

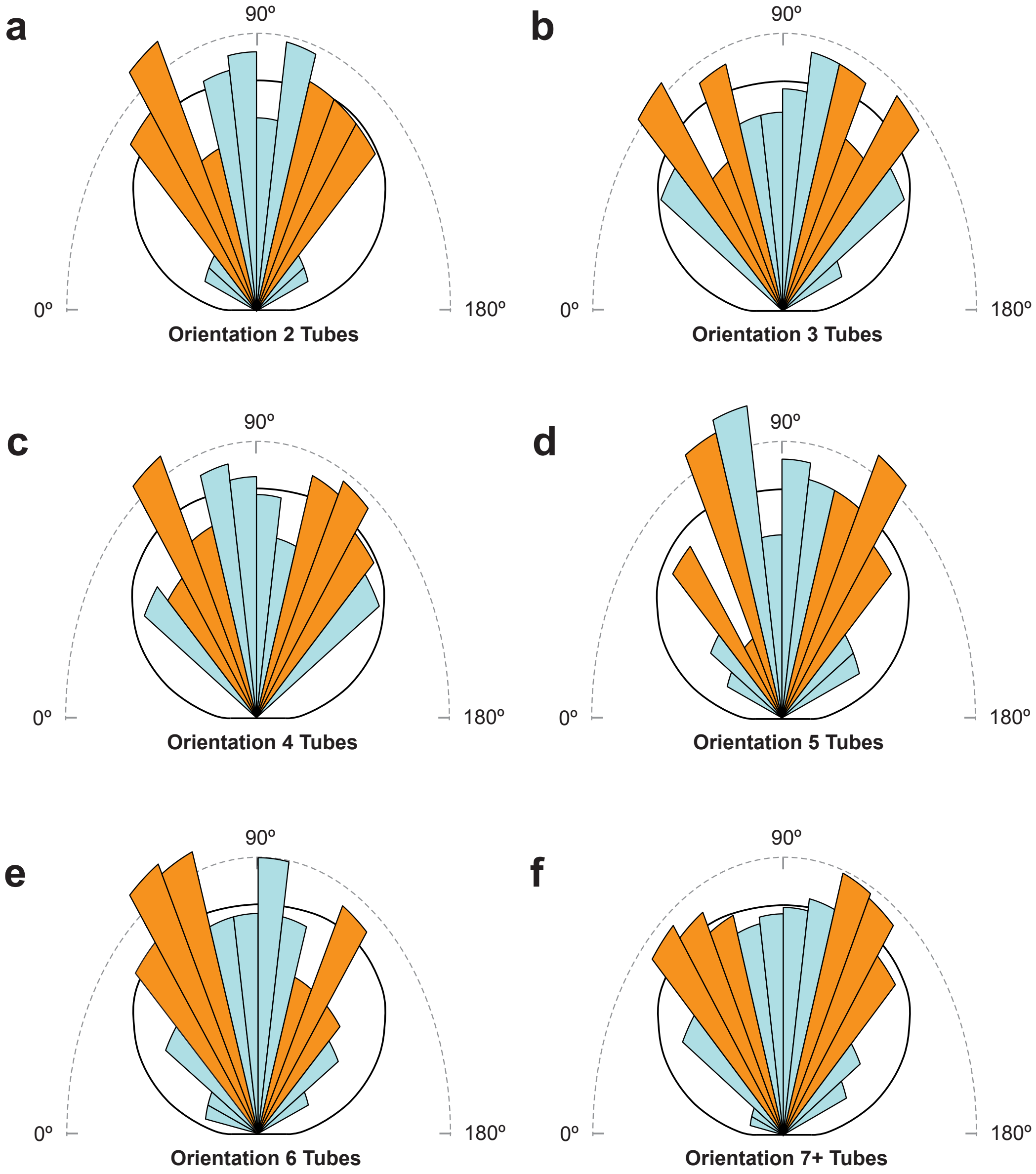

Supplementary Fig. 7. Additional rose diagrams of attached tube orientation for all values of attached tubes. Each division represents a 10° interval. Intervals coloured in orange are those that correspond to the inhalant laminar currents generated by *N. wulongqingensis*. Rose diagrams for all measured individuals and individuals with only one attached tube are provided in Fig. 3. The radii of each sector is equal to the square root of the relative frequencies of observations for each group. Absolute values for each sector are provided in Supplementary Data 4. **a,** Individuals with 2 attached tubes. **b,** Individuals with 3 attached tubes. **c,** Individuals with 4 attached tubes. **d,** Individuals with 5 attached tubes. **e,** Individuals with 6 attached tubes. **f,** Individuals with 7+ attached tubes.

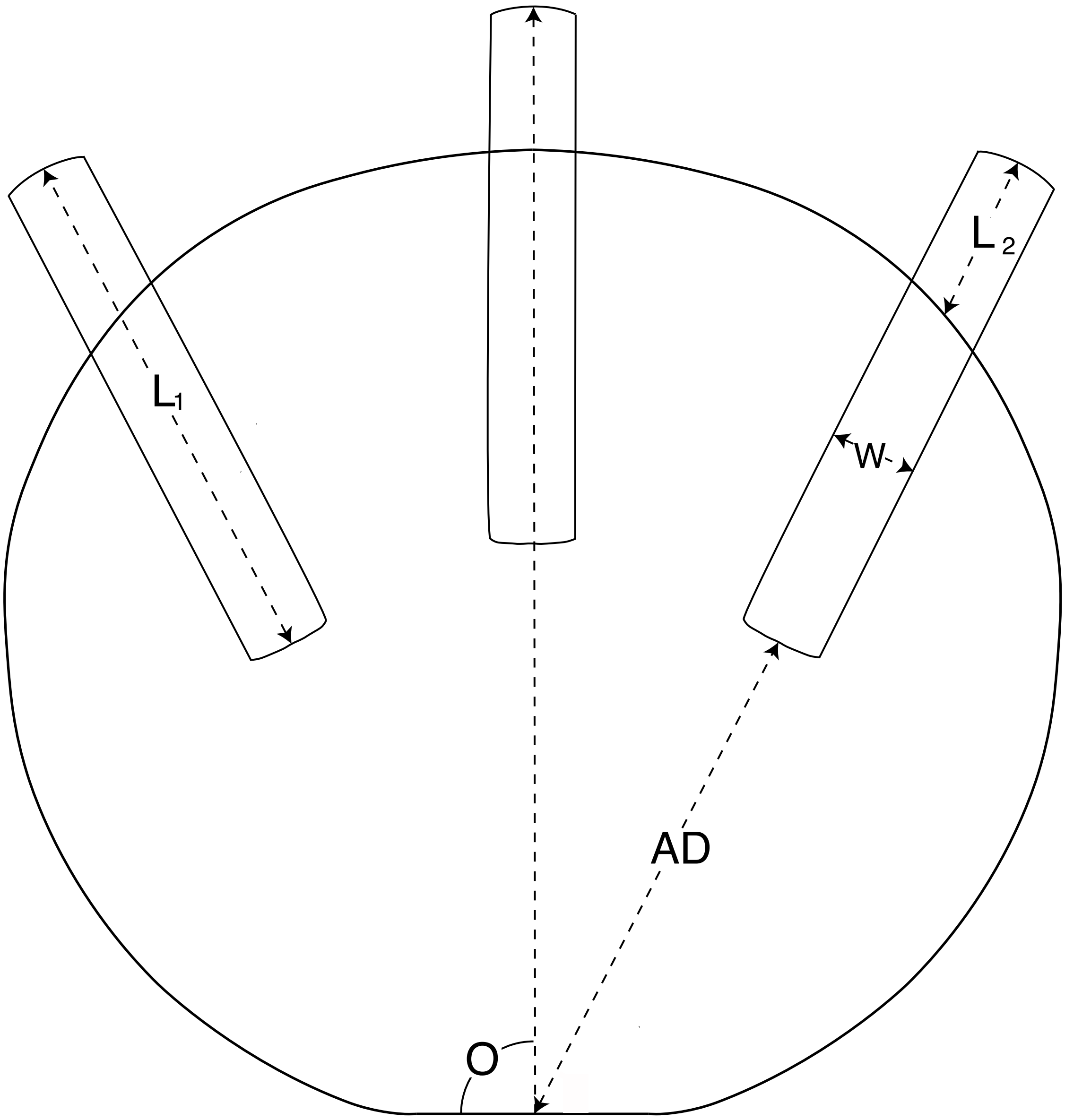

Supplementary Fig. 8. **Diagram showing methods used to take measurements.** Measurements include encrusted tube length (L1), tube length beyond anterior margin (L2), tube width (W), tube orientation (O) and tube attachment distance from posterior margin (AD).

Supplementary References

1. Chen, F.-Y., Zhang, Z.-F.., Betts, M. J., Zhang, Z.-L., Liu, F. First report on Guanshan Biota (Cambrian Stage 4) at the stratotype area of Wulongqing Formation in Malong County, Eastern Yunnan, China. *Geosci. Front.* **10**, 1459-1476 (2019).
