# Supplementary material for: An encrusting kleptoparasite-host interaction from the early Cambrian
Source: Nat Commun. 2020 Jun 2;11:2625. doi: 10.1038/s41467-020-16332-3 (PMC7266813; doi:10.1038/s41467-020-16332-3)
Supplement: Supplementary file 3 — Reporting Summary [file 41467_2020_16332_MOESM3_ESM.pdf]

nature research

Corresponding author(s): Zhifei Zhang, Luke Strotz, Timothy Topper

Last updated by author(s): 2020/04/20

Reporting Summary

Nature Research wishes to improve the reproducibility of the work that we publish. This form provides structure for consistency and transparency in reporting. For further information on Nature Research policies, see Authors & Referees and the Editorial Policy Checklist .

Statistics

For all statistical analyses, confirm that the following items are present in the figure legend, table legend, main text, or Methods section.

n/a	Confirmed	
☐	☒	The exact sample size (*n*) for each experimental group/condition, given as a discrete number and unit of measurement
☐	☒	A statement on whether measurements were taken from distinct samples or whether the same sample was measured repeatedly
☐	☒	The statistical test(s) used AND whether they are one- or two-sided<br>*Only common tests should be described solely by name; describe more complex techniques in the Methods section.*
☐	☒	A description of all covariates tested
☐	☒	A description of any assumptions or corrections, such as tests of normality and adjustment for multiple comparisons
☐	☒	A full description of the statistical parameters including central tendency (e.g. means) or other basic estimates (e.g. regression coefficient) AND variation (e.g. standard deviation) or associated estimates of uncertainty (e.g. confidence intervals)
☐	☒	For null hypothesis testing, the test statistic (e.g. *F*, *t*, *r*) with confidence intervals, effect sizes, degrees of freedom and *P* value noted<br>*Give P values as exact values whenever suitable.*
☐	☒	For Bayesian analysis, information on the choice of priors and Markov chain Monte Carlo settings
☒	☐	For hierarchical and complex designs, identification of the appropriate level for tests and full reporting of outcomes
☐	☒	Estimates of effect sizes (e.g. Cohen's *d*, Pearson's *r*), indicating how they were calculated

Our web collection on statistics for biologists contains articles on many of the points above.

Software and code

Policy information about availability of computer code

Data collection	Image J 1.49v
Data analysis	Performed in R 3.6.1 using standard packages outlined in the methods. No unique code was generated

For manuscripts utilizing custom algorithms or software that are central to the research but not yet described in published literature, software must be made available to editors/reviewers. We strongly encourage code deposition in a community repository (e.g. GitHub). See the Nature Research guidelines for submitting code & software for further information.

Data

Policy information about availability of data

All manuscripts must include a data availability statement. This statement should provide the following information, where applicable:

- Accession codes, unique identifiers, or web links for publicly available datasets
- A list of figures that have associated raw data
- A description of any restrictions on data availability

The data supporting the findings of this study are available in the paper and its supplementary information files or from the corresponding authors upon request. All specimens illustrated in this paper are reposited in the Early Life Institute (ELI), Northwest University, Xi'an, China.

Field-specific reporting

Please select the one below that is the best fit for your research. If you are not sure, read the appropriate sections before making your selection.

Ecological, evolutionary & environmental sciences study design

All studies must disclose on these points even when the disclosure is negative.

Study description

A study of a unique symbiotic relationship between a new brachiopod species and associated tube worms from the early Cambrian Guanshan Konservat-Lagerstätte in southern China. Using a suite of statistical techniques, we demonstrate a difference in organismal fitness exists between specimens with encrusted tube worms and those without. Further analyses demonstrates this relationship represents a form of kleptoparasitism. Total number of brachiopod specimens was N=429. All analyses use a single factor or variable (e.g. biomass, tube orientation etc).

Research sample

Fossil specimens for this palaeontological study were collected from slabs of fine grained mudrocks and siltstones from thin-bedded mudrock layers within the basal 20 m of the Wulongqing Formation (formerly Wulongqing Member of Canglangpu Formation) from the Gaoloufang section, near Kunming, China. The sample is representative and the samples investigated were chosen due to their exceptional preservation that would allow accurate and reliable measurements.

Sampling strategy

All specimens collected during the excavation were investigated and well preserved specimens that permitted accurate measurements were analysed. No statistical methods were used in the sampling of fossil material, but to the best of our knowledge, our datasets represents the largest fossil dataset to analyse a symbiotic relationship in the fossil record and consequently we deem the dataset to be sufficient.

Data collection

Specimens were photographed using a Zeiss Smart Zoom 5 Stereomicrographic system and micro-XRF elemental mapping was undertaken using a Bruker M4 Tornado Micro-XRF spectrometer. Measurements were made directly on photographs of specimens using ImageJ 1.49v. F-Y.C., Y-L.C., Y.L. photographed the specimens and completed EDX and micro-XRF analyses. T.P.T and Y.L. completed all the measurements. L.C.S. and Y-L.C. completed all statistical analyses. Collection of fossil specimens was made by a a group of masters and PhD students including F-Y.C., Y-L.C, Y.L.

Timing and spatial scale

Fossils were collected in 2014–2018 from stratigraphic horizons within the basal 20 m of the Wulongqing Formation from the Gaoloufang section, near Kunming, South China. Specimens were sampled from the basal 20 m of the Wulongqing Formation because that is where the fossils occurred. Fossils were collected during those listed field seasons as that is when funding and timing was sufficient to allow trips. After the 5 field seasons the quarry at the Gaolufang section was approximately 6 meters in width and over 800 brachiopods with encrusted tubes were collected.

Data exclusions

No data were excluded from the analysis.

Reproducibility

All the data and methods necessary to reproduce our results are provided in our main manuscript and the associated supplementary information. All specimens used in our study are accessioned in the Northwest University palaeontological collections and are accessible to all current and future researchers. Whilst experiments may be replicated in certain fields, this is impossible in palaeontology given only a single fossil record exists.

Randomization

Specimens were assigned to two groups for primary analysis: those with encrusted tubes and those without encrusted tubes

Blinding

Our study did not require blinding because it did not involve experiments.

Did the study involve field work? ☒ Yes ☐ No

Field work, collection and transport

Field conditions

The field site is located in the subtropical zone. Climate is humid in the summer field seasons and cold in the winter field seasons. According to climate-data.org, the average temperature of Kunming (the nearest large city) is 15.2 degrees with an annual rainfall of 999 mm. During the summer months, average temperatures rise to approximately 20 degrees and rainfall is between 180 and 210 mm per month. This decreases dramatically in the colder months that see less than 20 mm of rain per month. Outcrops are well exposed and easily accessible. Excavation was required to remove slab of fossil specimens for analysis, but excavation was done in a responsible manner.

Location

Located at Gaoloufang, Guangwej Villages in Kunming of Yunnan Province in South China, coordinates - N 2457'10''; E 10247'55''.

Access and import/export

Collection of fossil specimens was carried out in a responsible manner and in compliance with the local, national and international laws. Access to the field area was made by car as it is not far from a paved track and nearby villages. All specimens illustrated in this paper are deposited in the Early Life Institute (ELI), Northwest University, Xi'an, China. No importing or exporting of samples was necessary and samples were transported back to Northwest University by automobile.

Disturbance

In order to collect, quantities of rock had to be removed. As stated above the area of excavation was approximately 6 metres in width by the end of the 5 years of field season. Disturbance however, was minimized by controlled excavation in this single area. Reclamation of the quarries will be carried out after the research project is completed.

Reporting for specific materials, systems and methods

We require information from authors about some types of materials, experimental systems and methods used in many studies. Here, indicate whether each material, system or method listed is relevant to your study. If you are not sure if a list item applies to your research, read the appropriate section before selecting a response.

Materials & experimental systems

n/a	Involved in the study
☒	☐ Antibodies
☒	☐ Eukaryotic cell lines
☐	☒ Palaeontology
☒	☐ Animals and other organisms
☒	☐ Human research participants
☒	☐ Clinical data

Methods

n/a	Involved in the study
☒	☐ ChIP-seq
☒	☐ Flow cytometry
☒	☐ MRI-based neuroimaging

Palaeontology

Specimen provenance	Fossils were collected from stratigraphic horizons within the basal 20 m of the Wulongqing Formation from the Gaoloufang section, near Kunming, South China. Collection permits were not needed.
Specimen deposition	All specimens illustrated in this paper are reposited in the Early Life Institute (ELI), Northwest University, Xi'an, China.
Dating methods	Stratigraphic correlation with contemporaneous Cambrian strata in China and abroad are used to constrain the age of the fossils. Regarding the age of the biota no new data was presented in this study.

☐ Tick this box to confirm that the raw and calibrated dates are available in the paper or in Supplementary Information.
