# Supplementary material for: An encrusting kleptoparasite-host interaction from the early Cambrian
Source: Nat Commun. 2020 Jun 2;11:2625. doi: 10.1038/s41467-020-16332-3 (PMC7266813; doi:10.1038/s41467-020-16332-3)
Supplement: Supplementary file 4 — Description of Additional Supplementary Files [file 41467_2020_16332_MOESM4_ESM.pdf]

Description of Additional Supplementary Files

File Name: Supplementary Data 1

Description: Body size and other pertinent data for 429 individuals of *N. wulongqingensis* from the Guanshan Biota.

File Name: Supplementary Data 2

Description: Measurements of attached tube dimensions and other relevant characteristics for 186 specimens of *N. wulongqingensis* from the Guanshan Biota.

File Name: Supplementary Data 3

Description: Minima, maxima, median and bounds of box and whiskers for Supplementary figures 6a and 6d

File Name: Supplementary Data 4

Description: Distribution of tube orientations on the surface of *N. wulongqingensis* from the Guanshan Biota.
